# A case of takayasu arteritis presenting with multiple cerebral infarcts: Diagnostic and therapeutic challenges

**DOI:** 10.6026/973206300220071

**Published:** 2026-01-31

**Authors:** Saifullah Syed, Devishi Sarin, Waqas Ahmed, Syeda Zainab Hassan, Sami Mehyar, Ankita Vaghani, Muhammad Subhan

**Affiliations:** 1 Department of Medicine, Royal College of Surgeons in Ireland, Dublin, Ireland; 2 Department of Medicine, Fudan University, Shanghai, China; 3 Department of Medicine, Lancashire Teaching Hospitals, Chorley, England; 4 Department of Medicine, Jinnah Hospital, Lahore, Pakistan; 5 Department of Medicine, MISR University for Science and Technology, Giza, Egypt; 6 Department of Family Medicine, Obstetrics and Gynaecology, Vaghani Hospital and Maternity home- Cardiac and Diabetes center, Surat, India; 7 Department of Medicine, Jinnah hospital Lahore, Allama Iqbal Medical College, Lahore, Pakistan

**Keywords:** cerebrovascular accidents (CVA), digital subtraction angiography, giant cell arteritis (GCA), ischemic stroke, large-vessel vasculitis, MRI, multiple cerebral infarcts, smoking, smoking tobacco, takayasu arteritis

## Abstract

Takayasu arteritis (TA) is a rare chronic granulomatous vasculitis affecting the aorta and its major branches, with neurological complications such as multiple cerebral infarcts being unusual but potentially disabling. We describe the case of a 49-year-old male c hronic smoker who presented with acute lower-limb paralysis. Initial evaluation resulted in misdiagnosis, delaying definitive management. Neuroimaging revealed infarcts in the distal anterior cerebral artery and left posterior cerebral artery territories, while digital subtraction angiography demonstrated critical occlusions and stenoses of the carotid and vertebral arteries, confirming TA. The patient received corticosteroids, anticoagulants, and intensive physiotherapy, leading to significant neurological recovery. Thus, we show the importance of maintaining a high index of suspicion for TA in middle-aged patients with unexplained multiple cerebral infarcts and absent peripheral pulses. Early vascular imaging, prompt initiation of immunosuppressive therapy, and a multidisciplinary treatment approach are essential to improve outcomes and reduce long-term disability in such rare but high-risk presentations.

## Background:

Takayasu arteritis (TA) is a rare, chronic granulomatous vasculitis involving mainly the aorta and its large branches, frequently resulting in progressive arterial stenosis or occlusion [1, 2]. Neurological complications, such as ischemic stroke, can occur as a result of impaired cerebral perfusion and have been documented in as many as 20% of patients with TA [3]. However, such manifestations are frequently insidious and prone to misdiagnosis, especially in the absence of overt constitutional symptoms or classical vascular signs during the early stages of disease [3, 4]. Early diagnosis of severe acute cerebrovascular complications of TA is important since prompt initiation of immunosuppression can markedly decrease the risk of permanent neurological impairments [4, 5]. Sophisticated neuroimaging techniques, specifically diffusion-weighted imaging magnetic resonance imaging (DWI-MRI), are critical for detecting multifocal ischemic infarctions and separating nonatherosclerotic vasculopathies from typical stroke causes [6, 7]. We present a case of a middle-aged male patient with TA who had recurrent cerebral infarctions in different vascular territories. Therefore, it is of interest to focus the diagnostic pitfalls of unusual presentations of stroke and the need for correlating vascular and neuroimaging findings to make an early diagnosis and treatment of systemic vasculitides.

## Case presentation:

**Figure 1:**
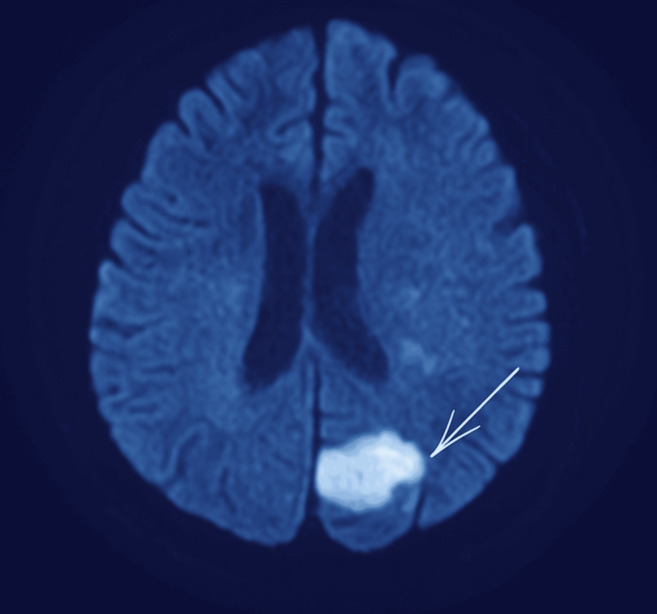
Brain MRI (Magnetic Resonance Imaging) with DWI showing left posterior cerebral artery infarct

**Figure 2:**
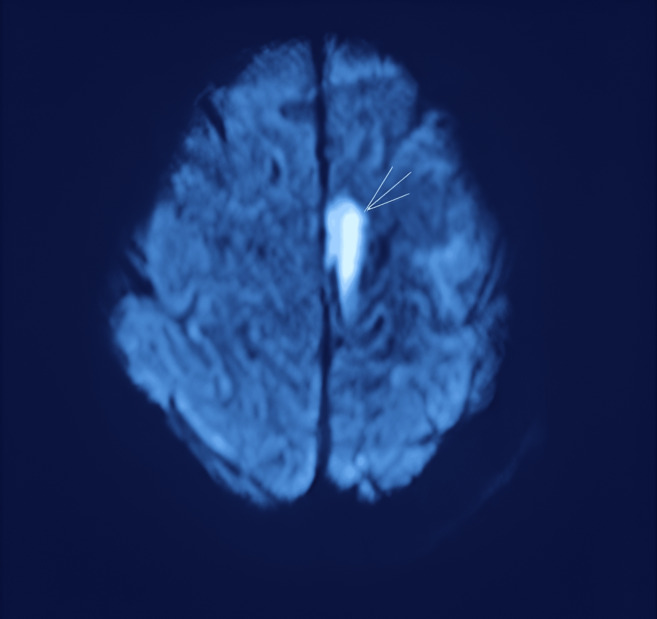
Brain MRI (Magnetic Resonance Imaging) DWI (Diffusion Weighted Imaging) showing anterior cerebral infarct territory

**Figure 3:**
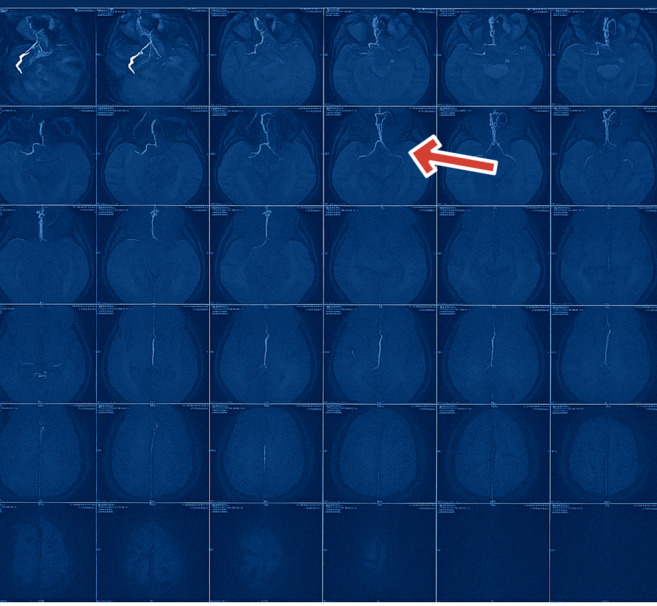
Magnetic Resonance Angiography (MRA) highlights the absence of visualization of the left Internal Carotid Artery (ICA), indicative of a possible occlusion or severe stenosis. The red arrow shows the absence of the left ICA.

**Figure 4:**
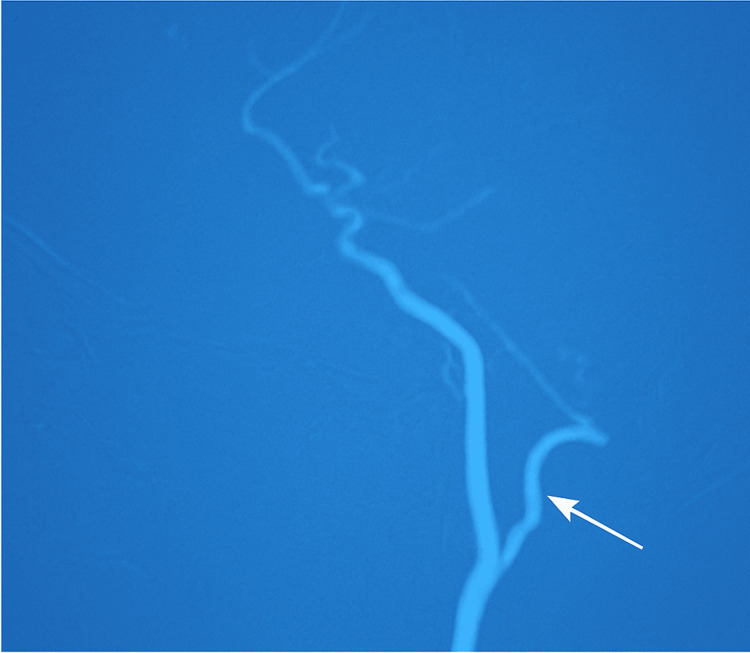
Digital Subtraction Angiography (DSA) demonstrates significant narrowing of the left vertebral arter

**Figure 5:**
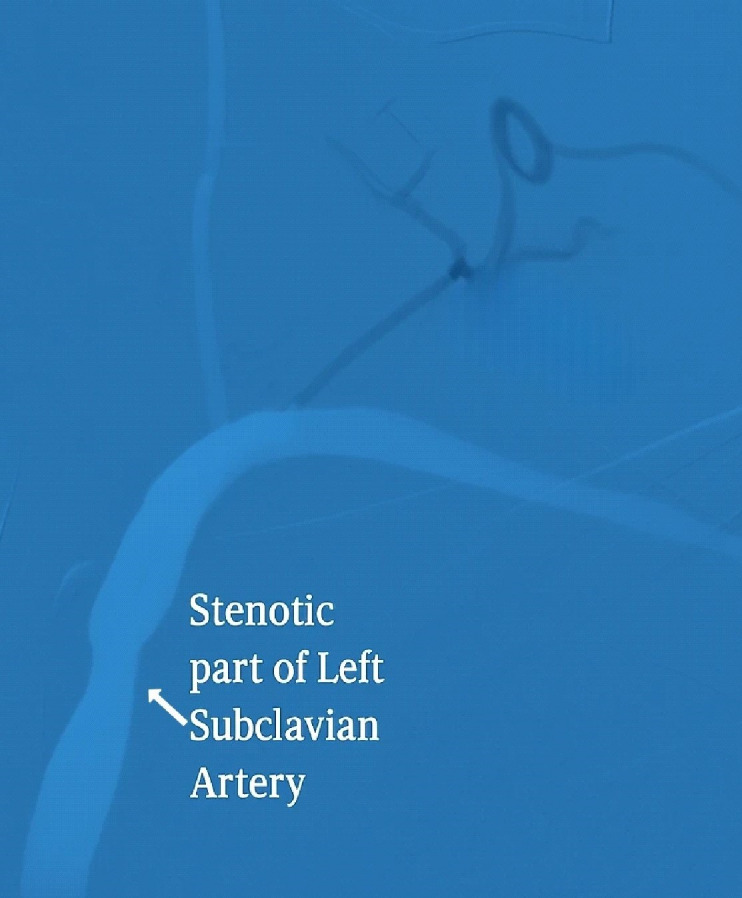
Digital Subtraction Angiography (DSA) shows pronounced stenosis of the left subclavian artery, potentially affecting blood flow to the upper limb and cerebral circulation.

A 49-year-old right-handed male, with a 20-pack-year smoking history and no prior diagnosis of hypertension, diabetes mellitus, dyslipidemia, or alcohol use, presented to our tertiary care facility with a four-week history of progressively worsening lower limb weakness and chronic occipital headaches. The headaches were dull, non-throbbing, and often associated with dizziness, visual blurring, and intermittent gait unsteadiness. Over the past two weeks, he also reported urinary urgency and occasional episodes of imbalance, prompting multiple visits to local clinics where he was treated symptomatically without sustained relief. On examination, he was alert, oriented, and afebrile. His blood pressure measured 150/90 mmHg in the right upper limb and 122/84 mmHg in the left. Notably, the left radial and brachial pulses were absent, while the right-sided peripheral pulses were palpable and symmetric. Femoral pulses were reduced bilaterally, and no audible bruits were heard over the carotid or subclavian regions. Cardiovascular and respiratory examinations were unremarkable. Neurological assessment revealed bilateral lower limb weakness with muscle power graded as 3/5 on the Medical Research Council (MRC) scale, while upper limb strength remained intact (5/5). Deep tendon reflexes of the lower limbs included hyperactive reflexes, with bilateral extensor plantar responses. The tone was normal across all extremities. Although there were no indications of cerebellar dysmetria or dysdiadochokinesia, the gait evaluation was noteworthy for a positive Romberg sign and noticeable unsteadiness on tandem walking. All modalities of sensory evaluation were unharmed, and cranial nerve function was maintained, allowing for a thorough assessment of the patient's neurological state. Brain MRI with DWI revealed multiple acute infarcts involving the left posterior cerebral artery (PCA) territory and the distal anterior cerebral artery (ACA) territory, as shown in Figure 1 and Figure 2. These lesions were hyperintense on DWI and correspondingly hypointense on apparent diffusion coefficient (ADC) mapping, consistent with acute ischemia. Magnetic resonance angiography demonstrated poor visualization of the left internal carotid artery (ICA) and suggested flow reduction in both vertebral arteries, as shown in Figure 3 Figure 4 shows the diagnostic digital subtraction angiography (DSA) that subsequently confirmed complete occlusion of the left vertebral artery, approximately 70% stenosis of the right vertebral artery, and significant stenosis of the bilateral common carotid arteries. Additional findings consistent with large-vessel vasculitis were uneven constriction of the left subclavian artery and branches of the aortic arch on DSA, as shown in Figure 5. Table 1 shows the summary of the important laboratory test results with their interpretation. Rheumatoid factor, antiphospholipid antibody panel, and viral serologies for HIV, hepatitis B and hepatitis C were nonreactive.

Electrocardiography and transthoracic echocardiography ruled out cardiac sources of embolism and showed no structural abnormalities or valvular lesions. In the absence of common atherosclerotic risk factors and with characteristic vascular involvement on angiography, a diagnosis of TA was established based on the 1990 American College of Rheumatology (ACR) criteria, with five out of six criteria fulfilled: onset before 60 years of age, claudication of extremities, decreased brachial pulse, inter-arm systolic BP difference >10 mmHg, and angiographic evidence of arterial narrowing consistent with large-vessel vasculitis. Initial management included subcutaneous enoxaparin at a dose of 60 mg twice daily (1 mg/kg), later transitioned to oral apixaban 5 mg twice daily for secondary stroke prevention. Given the inflammatory etiology, immunosuppressive therapy was initiated with oral prednisolone at 60 mg/day (1 mg/kg/day), in accordance with the European Alliance of Associations for Rheumatology (EULAR) 2018 recommendations, which endorse high-dose glucocorticoids as first-line therapy for Takayasu arteritis, followed by gradual tapering. To facilitate steroid-sparing and achieve long-term disease control, oral methotrexate at 15 mg/week with folic acid supplementation was introduced on day seven, consistent with guideline-supported use of conventional disease-modifying antirheumatic drugs (DMARDs) in large-vessel vasculitis. Low-dose aspirin (75 mg/day) was continued, aligning with evidence that antiplatelet therapy may reduce ischemic events in patients with large-vessel vasculitis, although high-quality randomized data are limited. The patient was also referred to physiotherapy, where a focused regimen on gait training and strength recovery was implemented, reflecting recommendations that early rehabilitation is essential to maximize neurological recovery following stroke. By the sixth hospital day, the patient showed marked improvement in lower-limb power (4/5), improved coordination, and was able to ambulate with minimal assistance, demonstrating the benefit of a multidisciplinary and evidence-based treatment strategy. A vascular surgery consultation was sought to consider elective stenting of the right vertebral artery and left carotid system; however, the patient declined invasive intervention at that time. He was discharged in the second week in stable condition, with follow-up planned in rheumatology and neurology clinics. The patient was still completely ambulatory and neurologically stable and complied with immunosuppressive treatment at his monthly follow-up appointment. Follow-up MRA revealed stable arterial stenoses without advancement, and follow-up inflammatory indicators had returned to normal. Table 2 shows the clinical timeline of the patient's presentation, investigations, and management.

## Discussion:

This case emphasizes the clinical diagnostic and therapeutic challenges of TA. This uncommon but severe vasculitis can present atypically, especially when initial symptoms involve neurological deficits rather than classic constitutional signs [1]. We reported the case of a 49-year-old male with multifocal cerebral infarctions who was ultimately diagnosed with TA based on clinical presentation and advanced imaging. The purpose of this case is to highlight how crucial it is to keep a high index of suspicion for TA when stroke presentations deviate from standard vascular risk profiles, especially when peripheral pulses are absent. DWI MRI validated our observations of infarcts in the ACA and PCA areas. These lesions occurred in the absence of traditional stroke risk factors such as atrial fibrillation, significant atherosclerosis, diabetes, or hypertension [2]. MRA and DSA further revealed bilateral vertebral artery disease, occlusion of the left internal carotid artery, and >70% stenosis in the right vertebral artery. Clinically, the absent left radial pulse, significant inter-arm blood pressure difference (>10 mmHg), and angiographic evidence of large-vessel involvement satisfied 5 out of 6 ACR 1990 diagnostic criteria for TA. These results underscore the need for extensive vascular imaging in patients with unexplained multifocal infarction when signs of peripheral vascular involvement are present. These findings are significant in reemphasizing the atypical presentation of TA. Although TA typically occurs among women younger than 40, recent data indicate that as many as 20% of the patients are over this age range [3]. The neurological involvement in TA, including stroke, develops in around 10-20% of cases, usually as a result of stenotic or occlusive lesions of the aortic arch and major vessels [4]. The vertebrobasilar circulation was mainly involved in our patient, which led to the posterior infarctions. Combining clinical findings (e.g., pulse abnormalities) with imaging studies cannot be overemphasized, as early recognition is necessary to avoid irreversible ischemic injury. Recent reports have emphasized vertebral artery involvement-whether stenosis, occlusion, or aneurysm-as a significant risk marker for neurological complications in TA [5,6-7]. Su *et al.* (2022) described aneurysmal dilation and occlusion of the cervical vertebral artery in a TA patient, underscoring vertebral artery fragility in the disease [5]. Zhang *et al.* (2023) found that vertebral artery lesions were prevalent in nearly one-third of TA-associated strokes in their cohort [6]. Our case is remarkable because of the patient's sex, age, and isolated neurological involvement without systemic signs. Other contributions have also been reported; for example, Moisii *et al.* (2024) reported delayed diagnosis due to non-specific symptoms, and usually, the patient reaches the stage with irreversible sequelae 7]. In contrast, our case highlights the importance of early multimodal imaging and diagnostic suspicion based on vascular exams. From a clinical point of view, early immunosuppressive treatment is imperative in cases like this one. We started oral prednisolone at 1 mg/kg/day (daily dose of 60 mg), which was gradually tapered over 12 weeks. Methotrexate was added at 15 mg/week as a steroid-sparing agent, by the European Alliance of Associations for Rheumatology (EULAR) and American College of Rheumatology (ACR) recommendations for the treatment of large-vessel vasculitis [8,9-10]. Due to the presence of acute cerebral infarction and severe 8 of 10 arterial narrowing, anticoagulation was initiated with subcutaneous enoxaparin (1 mg/kg BID) and later transitioned to oral apixaban (5 mg BID). The patient refused carotid stenting because of economic reasons. Nonetheless, his neurological symptoms improved dramatically with pharmacotherapy alone, and inflammatory markers returned to normal at 6 weeks. Mobility was also enhanced with rehabilitation and physical therapy, emphasizing the value of supportive care. Its clinical implications are profound. It demonstrates the need to expand the differential diagnosis of stroke in atypical patients and to consider large-vessel vasculitis in patients with multifocal infarctions and pulse abnormalities. Furthermore, it supports early and aggressive immunosuppression, even without systemic signs, to control vascular inflammation and improve outcomes. This case also adds to the limited literature on TA presenting primarily with neurologic symptoms in older males, expanding the demographic understanding of the disease. This single-patient case limits generalizability. Mildly elevated ESR and CRP emphasize the low efficacy of common markers in cerebral-predominant Takayasu arteritis, and new biomarkers are required. The patient denied invasive treatment options. Other aspects to be addressed in the future include predictive factors, comparison among medical and interventional treatments, and cost effectiveness of advanced diagnostics in a low-resource scenario. In patients with stroke, pulse deficit, or inter-arm BP differences, Takayasu arteritis should be considered. Early vascular imaging and timely immunosuppression can improve outcomes, even with regular inflammatory markers.

## Conclusion:

TA should be used to diagnose patients with recurrent cerebral infarcts differentially, particularly when there are missing pulses or inter-arm blood pressure differences. Although classically occurring in young women, this case is a reminder that it can happen in older men and that, accordingly, there should be heightened clinical suspicion in atypical presentations. Early use of MRI, MRA, and DSA allowed for timely diagnosis, and early treatment with corticosteroids, anticoagulants and immunosuppressants had a favorable outcome. Multidisciplinary management is necessary for optimal care and long-term follow-up of such complicated cases.

## Figures and Tables

**Table 1:** Laboratory Investigations

**Test**	**Result**	**Reference Range**	**Interpretation**
Hemoglobin (Hb)	14.2 g/dL	13.5 - 17.5 g/dL (Males)	Normal
Total Leukocyte Count (TLC)	7,500 /µL	4,000 - 11,000 /µL	Normal
Platelet Count	280,000 /µL	150,000 - 450,000 /µL	Normal
Erythrocyte Sedimentation Rate (ESR)	24 mm/hr	< 20 mm/hr	Mildly Elevated (suggests inflammation)
C-Reactive Protein (CRP)	7.2 mg/L	< 6 mg/L	Elevated (acute phase reactant)
ANA (Antinuclear Antibody)	Negative	Negative	Normal
ANCA (Anti-Neutrophil Cytoplasmic Ab)	Negative	Negative	Normal
AST (Aspartate Transaminase)	26 U/L	10 - 40 U/L	Normal
ALT (Alanine Transaminase)	30 U/L	7 - 56 U/L	Normal
Serum Creatinine	0.9 mg/dL	0.6 - 1.2 mg/dL	Normal
Blood Urea	28 mg/dL	7 - 20 mg/dL	Mildly Elevated (non-specific)
Serum Total Cholesterol	178 mg/dL	< 200 mg/dL	Normal
HDL Cholesterol	50 mg/dL	> 40 mg/dL	Normal
LDL Cholesterol	100 mg/dL	< 130 mg/dL	Normal
ECG	Normal	-	No arrhythmia or ischemia noted
Echocardiogram	Normal	-	Normal cardiac structure and function

**Table 2:** Clinical Timeline of the Patient’s Presentation and Management BP: Blood Pressure, MRI: Magnetic Resonance Imaging, MRA: Magnetic Resonance Angiography, PCA: Posterior Cerebral Artery, ACA: Anterior Cerebral Artery, DSA: Digital Subtraction Angiography

**Date / Duration**	**Event / Clinical Finding**
Week -4 to -1	Gradual onset of bilateral lower limb weakness and chronic headache
Day 0	Presentation to our institution with immobility and imbalance
Day 1	Physical exam: BP 150/90 mmHg, absent left radial pulse
Day 2	MRI Brain + MRA: Infarcts in left PCA and distal ACA territory
Day 3	DSA: Bilateral carotid stenosis, left vertebral artery occlusion
Day 4	Initiated anticoagulation + low-dose corticosteroids + physiotherapy
Day 6	Improvement: able to stand with support
Day 10	Further improvement in mobility, headache reduced
Week 2	Diagnosis of Takayasu arteritis confirmed (clinical + radiological)
Month 1 (Follow-up)	Stable condition, continued improvement, declined carotid stenting
